# Assisted reproduction technology and long-term cardiometabolic health in the offspring

**DOI:** 10.1371/journal.pmed.1003724

**Published:** 2021-09-07

**Authors:** Ronald C. W. Ma, Noel Y. H. Ng, Lai Ping Cheung

**Affiliations:** 1 Department of Medicine and Therapeutics, The Chinese University of Hong Kong, Hong Kong; 2 Li Ka Shing Institute of Health Sciences, The Chinese University of Hong Kong, Hong Kong; 3 Hong Kong Institute of Diabetes and Obesity, The Chinese University of Hong Kong, Hong Kong; 4 Department of Obstetrics and Gynaecology, The Chinese University of Hong Kong, Prince of Wales Hospital, Shatin, New Territories Hong Kong

## Abstract

Ronald Ma and co-authors discuss Emma Norrman and colleagues’ accompanying research study on the health of children born with assisted reproductive technology.

Since the birth of the first baby with the aid of in vitro fertilization (IVF) in July 1978, more than 9 million children have since been born through IVF or other assisted reproduction technology (ART). From a report covering around 2/3 of world ART activity, it was estimated that more than 4.4 million ART cycles have been initiated between 2008 and 2010, which resulted in 1.14 million births during that period [1]. From 1997 to 2016, the numbers of recorded ART treatment have increased by 5.3-fold in Europe, 4.6-fold in the United States of America, and 3.0-fold in Australia and New Zealand [1]. In an accompanying study in *PLOS Medicine*, Emma Norrman and colleagues address the health of babies born after ART [2].

While initially met with considerable skepticism and controversy, the large number of healthy babies born over the last 4 decades is testament to the success and safety of IVF, which has transformed the lives of many couples and families. Nevertheless, given the appreciation of the Developmental Origins of Health and Disease (DOHaD) hypothesis, which posits that insults during critical times of development (including in utero or early life) may modify an individual’s phenotype and alter later risk of disease in adulthood, as well as previous demonstration of potential epigenetic changes following ART, there has been rekindled interest in the potential long-term effects of ART on offspring health [3]. In a large retrospective Nordic population-based cohort study of all children born after ART between 1982 and 2007, there was no significant increase in overall cancer rates among children born after ART, compared to children born after spontaneous conception (SC) [4]. Questions have also been raised about the long-term cardiovascular health of offspring born after ART, as several mechanisms have been postulated to potentially contribute to impaired cardiovascular health, including suboptimal culture conditions, ART-induced epigenetic changes, as well as indirect effects through low birthweight, thereby contributing to altered cardiovascular phenotype [5] (**Fig 1**). A systematic review and meta-analysis did not show evidence of increased cardiovascular risk or diabetes for women following ART, though there was comparatively less data to address offspring risk [6]. In line with a recent systematic review and meta-analysis [7], previous small studies have found increased adiposity, cardiometabolic risk, and blood pressure among offspring born after ART, potentially due to altered gene expression [8,9]. However, issues regarding potential selection bias have been raised for the small studies included.

**Fig 1:**
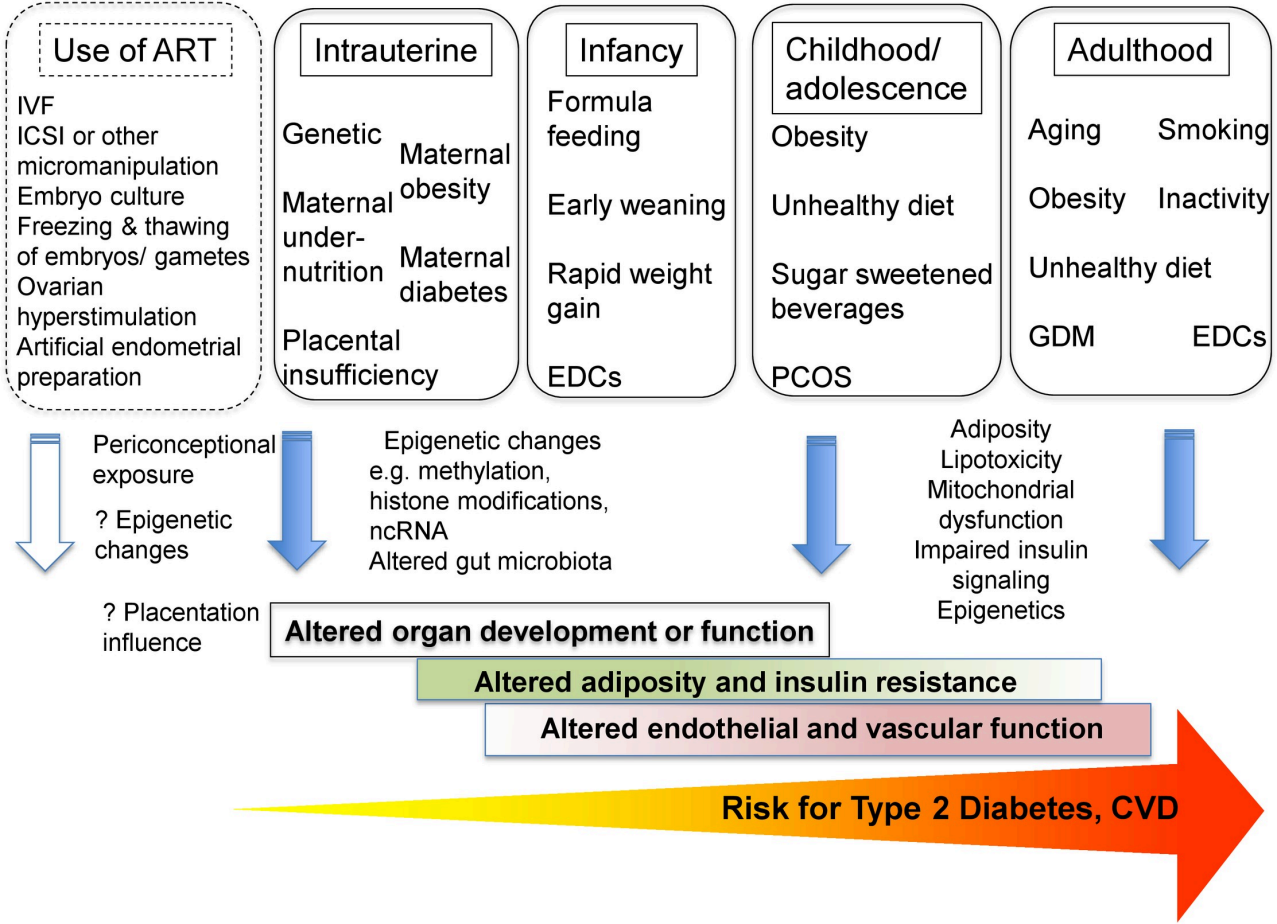
A DOHaD perspective on the potential relationship between ART and later risk of T2D and CVD. The putative link is highlighted by the dotted outline. ART, assisted reproduction technology; CVD, cardiovascular disease; DOHaD, Developmental Origins of Health and Disease; EDCs, endocrine-disrupting chemicals; GDM, gestational diabetes; ICSI, intracytoplasmic sperm injection; IVF, in vitro fertilization; ncRNA, noncoding RNA; PCOS, polycystic ovary syndrome; T2D, type 2 diabetes. (adapted with permission from Ma and colleagues [13]).

Norrman and colleagues conducted a large population-based study from the Committee of Nordic ART and Safety (CoNARTaS) cohort, which included all individuals born in Norway, Sweden, Finland, and Denmark between 1984 and 2015, including 122,429 children born after ART, and more than 7.5 million children born after SC, to investigate the risk of cardiovascular disease (CVD), diabetes, and obesity following ART compared to SC. Offspring were followed for a mean 8.6 years in children born after ART and 14.0 years for children born following SC. Although the crude rates for CVD and type 2 diabetes (T2D) were higher among children born after ART, there were no significant difference in rates after adjustment for measured confounders. The study noted a significant increase in the risk of obesity among children born to ART, though the risk was modest, with adjusted HR 1.14 (CI 1.06 to 1.23, *p* = 0.001). The design of the study also meant that it could not address whether any increased risk in the offspring might be related to maternal causes of infertility (such as polycystic ovary syndrome), rather than the ART. In contrast to the previous systematic review that suggested significant increase in cardiometabolic risk factors in ART offspring, the authors concluded that the cardiometabolic outcomes in ART children are, in general, reassuring. However, further studies with longer follow-up are needed.

The study provided much-needed medium-term outcome data addressing this important question of long-term cardiometabolic risk in children born after ART. By combining high-quality Nordic registers, Norrman and colleagues have been able to create a uniquely large cohort of ART children in order to compare their risk of cardiovascular health with children born after SC. Such population-based design provided high coverage rate and high validity, such that missing data and the risk of selection bias can be minimized. However, there were some notable limitations of the study, including the relatively short follow-up period, especially among children born to ART. The number of clinical outcomes of interest was limited, hence restricting statistical power to detect differences in outcome. Although the use of national registries has minimized any risk of selection bias, the definitions of outcomes were based on inpatient and outpatient attendance and may be associated with some ascertainment bias, especially in relation to capturing obesity outcomes. There is also a significant proportion with missing maternal BMI, paternal characteristics, or other covariates, which posed limitations on the analyses. Another important point to note is that the impact of different ART factors on health of offspring has not been addressed. Over the years, ART practices and technologies have continued to evolve, for example, the increasing use of oocyte freezing and embryo biopsy for genetic testing and the shift of slow freezing to vitrification method for gamete or embryo freezing. It has been shown that singleton babies conceived from fresh embryo transfers of IVF are associated with increased risks of low birthweight and preterm delivery, while ART involving frozen embryos are associated with higher incidences of large babies, macrosomia, and hypertensive disorders of pregnancy [10]. Conversely, both low birthweight, intrauterine growth restriction (IUGR), as well as macrosomia have been linked with increased risk of later T2D and CVD [11]. These differential outcomes illustrate that the different ART techniques may have different safety profiles and exert different impacts on the long-term health of offspring. Of note, the study by Norrman and colleagues included relatively few births from frozen embryos, and these have not been compared to births by SC for later risk of diabetes or CVD.

This important study highlights some of the challenges in ascertaining long-term effects of ART, or other early life exposures, for that matter. The establishment of ART registries including the important exposure factors may be an important component for the way forward, especially given the long-term follow-up required. There are important challenges, including those around confidentiality, but also the increasingly diverse and complex treatment protocols, as well as innovative technologies, which may be associated with different long-term outcomes. There is currently limited understanding of the long-term outcome of some of these novel techniques. There are also new challenges given the globalization of healthcare delivery, with increasing cross-border reproductive care [12]. The increasing cryopreservation of gametes, gonadal tissues, and embryos will pose new challenges on tracking the outcomes of ART births. More population-based long-term studies are warranted, and establishing the infrastructure that would facilitate anonymous linkage of ART registers, birth records with national diabetes and other disease registers that facilitate tracking of long-term health may be one way forward. Nevertheless, given the sensitivities around the data involved, such analyses may be difficult to perform in some areas, and population-based analyses, wherever possible, will continue to contribute much-needed data to this discussion.

## Abbreviations

ART: assisted reproduction technology
CoNARTaS: Committee of Nordic ART and Safety
CVD: cardiovascular disease
DOHaD: Developmental Origins of Health and Disease
IUGR: intrauterine growth restriction
IVF: in vitro fertilization
SC: spontaneous conception
T2D: type 2 diabetes
